# Inferior Oblique Weakening and Abnormal Head Position: Controlled Myotomy versus Recession

**DOI:** 10.1155/2016/1725484

**Published:** 2016-11-27

**Authors:** R. Migliorini, R. Malagola, A. M. Comberiati, L. Arrico

**Affiliations:** Department of Sense Organs, “Sapienza” University of Rome, Rome, Italy

## Abstract

*Purpose*. Randomized controlled trial aimed at comparing surgical outcomes in a group of patients suffering from hyperfunction of the inferior oblique (IO) muscle with abnormal head position (AHP). The surgical techniques being compared are Recession and (thread) Controlled Myotomy.* Materials and Methods*. The group of 20 patients suffering from medium-high hyperfunction of the IO was assessed through an ophthalmological and orthoptic examination. 10 patients underwent traditional Recession (Group  A) and 10 were treated with Controlled Myotomy (Group  B).* Results*. The average age was 19 years ± 10.7 SD. After 1 year, 20% of Group  A showed a small Vertical Deviation associated with a small AHP, while 80% had orthophoria and 40% of them had a small AHP. 80% of Group  B showed a small Vertical Deviation associated with an equally small AHP, while 20% had orthophoria with a full resolution of AHP.* Conclusion*. Based on the results obtained and the fewer intrasurgical risks involved, thread Controlled Myotomy proved to be a valid alternative to Recession. Furthermore, in case of Recession, over the long period a small residual AHP remained in the patients who had orthophoria, unlike Myotomy which led to a total resolution.

## 1. Introduction

Of the four cyclovertical muscles, the inferior oblique (IO) muscle is the one which is most frequently the object of a surgical therapy. In general, IO muscle surgery is limited to weakening procedures as this muscle is rarely paralyzed or so weakened as to require support procedures as discussed elsewhere [1–3]. Over the last decades, many authors have been studying this surgical approach and after years of experiences, at present, the most used techniques are those which proved to be the most effective in terms of utility and validity. In spite of the fact that, as shown in the literature, many techniques have been used, from the Tenotomy of the IO to Marginal Myotomy as discussed elsewhere [4, 5], the Recession of insertion described by White (1942) and later by Guibor (1944) [6] is considered to be the most final and accurate means of weakening, and in 1950 it became the most accurate procedure to eliminate the hyperfunction of the IO. In spite of being considered by many as technically harder that the others, if accurately performed, this technique may offer a satisfactorily predictable result.

Subsequently, as described by Pannarale and Leonardi (1978) [7], the surgical technique of Controlled Myotomy was presented using 2 different methods, depending on the material used:* plastic sling* or* plastic bridge sutures.*

**Figure 1:**
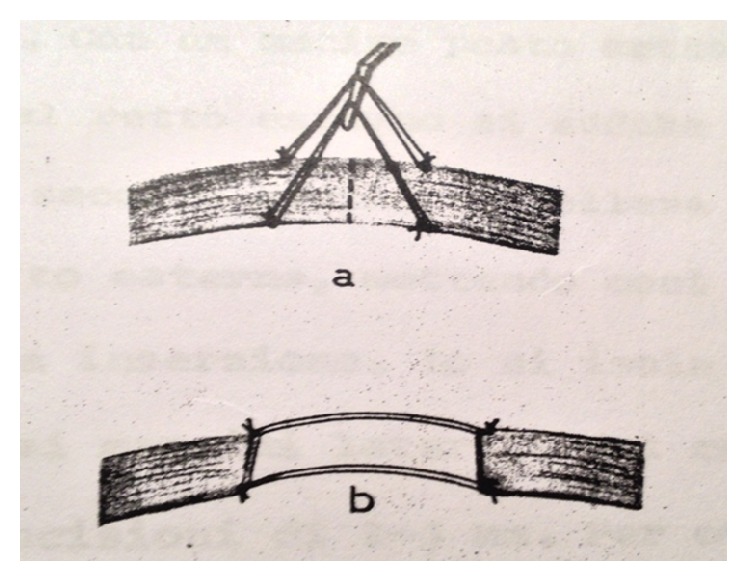
Controlled Myotomy by plastic bridge sutures.

With the second technique, the more used of the two, 2 nonreabsorbable suture threads (Ti-Cron 6-0) are placed directly on the muscle. The muscle is then cut following the pattern shown in Figure 1 which results in a weakening equal to the length of the threads.

The various techniques used to weaken the IO muscle are still raising conflicting opinions among the authors as to their effectiveness. Some surgeons, although considering Recession as discussed elsewhere [8–12], as the best surgical technique able to assure reliable results, preferred Myotomy Controlled by bridge sutures for its quick execution and low risk of intrasurgical complications. As a matter of fact, this latter technique allows to reduce the risk of possible hemorrhages, to prevent any accidental perforation of the globe, to respect the physiological scleral intersection point, and above all to adjust the level of weakening in the postsurgical period.

Based on these surgical considerations as discussed elsewhere [13–16], we have compared the surgical results obtained in two groups of patients suffering from hyperfunction of the IO muscle with abnormal head position (AHP). The two techniques compared were Recession and Myotomy Controlled through bridge sutures.

## 2. Materials and Methods

A group of 20 patients followed at the Pediatric Ophthalmology and Strabology of the Ophthalmology Department, Umberto I General Hospital in Rome, were randomly recruited. They were all suffering from a Vertical Deviation resulting from the hyperfunction of the inferior oblique muscle with AHP.

The patients underwent surgery aimed at weakening the inferior oblique muscle:

- number 10 through traditional Recession (Group  A);
- number 10 trough Myotomy Controlled with bridge nonreabsorbable sutures (Group  B).

After an accurate examination of their histories, the patients underwent a thorough ophthalmologic examination and were then subjected to the following tests: AHP assessment, examination of Corneal Reflexes, Cover Test, study of Extrinsic Ocular Motility, Bielschowsky Test in presence of a deficit of the Superior Oblique muscle, Red Glass Test in presence of diplopia, and Hess-Lancaster Screen test. Each patient was assessed by the same examiner in order to reduce any experimental prejudice.

The tests were repeated after 1 month and 1 year.

Inclusion criteria were

(i) age between 7 and 42;
(ii) patients who, at an accurate assessment of their Ocular Motility, showed a hyperfunction of the inferior oblique muscle ascribable to a hypofunction of the Superior Oblique muscle (homolateral antagonist) and/or hypofunction of the Superior Rectus (contralateral synergist);
(iii) patients with an evident abnormal head position (AHP);
(iv) patients with medium-high Vertical Deviation (≥10^DP^);
(v) patients who never underwent any surgical intervention to weaken the inferior oblique.

Exclusion criteria were

- visual acuity < 1/10 (Snellen Optotype);
- patients suffering from postsurgery relapses due to previous surgical weakening of the inferior oblique;
- patients for whom the Prismatic Adaptation Test (PAT) showed diplopia after 30–60 minutes.

In this controlled randomized pilot study, the two techniques were compared with Person comparison analysis in order to analyze whether Controlled Myotomy can give postsurgical results almost identical to those of Recession which so far has been considered the most used technique both in terms of accuracy of the execution and of prognosis predictability.

## 3. Results

**Figure 2:**
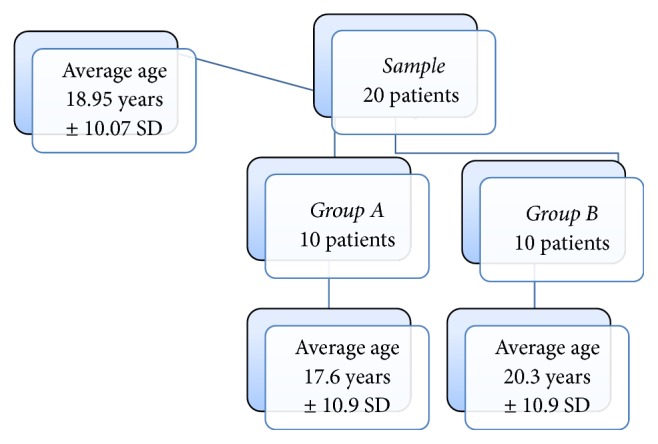
Stratification of the sample by age.

The average age of the entire sample was 19 years ± 10.07 SD; for Group  A it was 18 years ± 10.9 SD and for Group  B was 20 years ± 10.9 SD (Figure 2).

The Cover Test evidenced that all individuals had a Vertical Deviation in the primary gaze position, which was very evident from far and negligible at a short distance. The prevailing aspect observed was the hyperfunction of the inferior oblique on the hypofunction ipsilateral Superior Oblique (100% of cases were Bielschowsky Test positive). All the patient showed a medium-high (≥10^DP^) hyperfunction of the inferior oblique muscle and the average of the interventions on a single eye was 7 ± 2 SD both for Recession and for suture-Controlled Myotomy.

**Figure 3:**
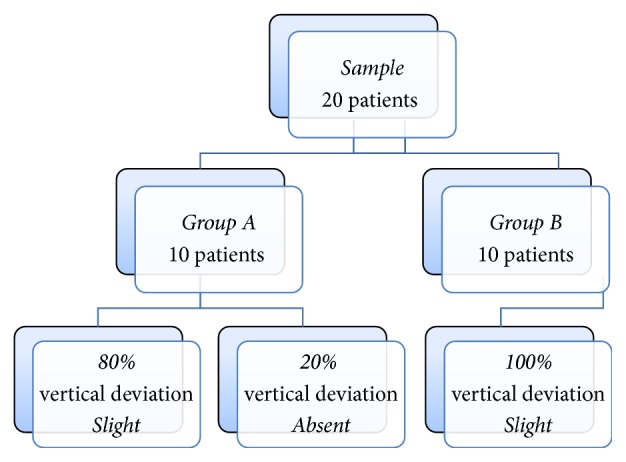
Follow-up at 1 month after operation.

One month after surgery, 80% of Group  A patients had a slight Vertical Deviation while the remaining 20% had orthophoria. Conversely, Group  B patients, always after 1 month, had—in 100% of the cases—a slight Vertical Deviation (Figure 3). Both in Recession and in Controlled Myotomy, all the patients still had an AHP one month after surgery.

**Figure 4:**
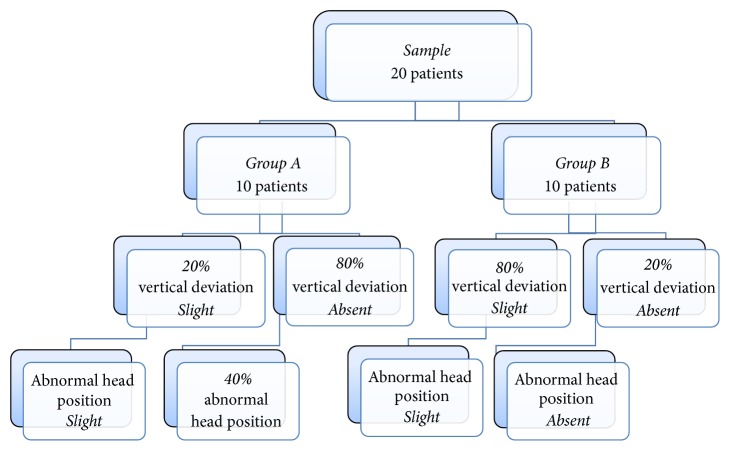
Follow-up at 1 year after operation.

One year after surgery, 80% of Group  A patients had a slight orthophoria and 40% a slight AHP, while 20% had a slight Vertical Deviation and a slight AHP. AHP was totally absent in 20% of Group  B patients (Figure 4). Pearson Correlation appeared moderate both 1 month and 1 year after surgery with a coefficient of 0.47.

## 4. Discussion

It was evident already after 1 year that Controlled Myotomy (Group  B) proved to be more effective in eliminating AHP. Indeed, in Group  B, a Slight Deviation still remained in 80% of the patients with the presence of a slight AHP, while in the remaining 20% orthophoria was obtained with a complete resolution of AHP while in Recession (Group  A) only 20% still had a slight Vertical Deviation with the presence of a slight AHP and in the 80% of the cases showed that the deviation was fully solved, but AHP was eliminated only in 40% of the patients. This result is easily ascribable to the opportunity offered by Controlled Myotomy to leave muscular insertion unchanged, thus contributing, in sensory terms, to a better recovery of the physiological spatial orientation. On the other hand, so far, Recession has been generally considered the most used technique both in terms of accuracy of the execution and of prognosis predictability, but we must also consider the complications such as antielevation and adherence syndrome that can develop after unilateral IO Recession surgery. Care should be taken not to tighten the neurofibrovascular bundle of the IO muscle during surgery as discussed by Niyaz et al. [17]. Recession is, according to many authors, the best method of inferior oblique muscle weakening and according to Parks [18] is dominant compared to all other methods. During and after surgery, in spite of procedure, the possible surgery and postoperative complications must be considered. Some of them are intraoperative and postoperative bleeding and hematoma in orbit, “adherent syndrome,” postoperative and continued hyperfunction of IO, hypotropia, internal ophthalmoplegia, and so on as discussed elsewhere [19, 20]. Instead, thread Controlled Myotomy does not provide all of these complications. Also in the literature, we did not find any other studies comparing the effectiveness of these two surgical techniques (Recession versus thread Controlled Myotomy). So this pilot study, despite the small number of participants in each group, for the good results achieved with Controlled Myotomy, is a small contribution that can change clinical practice.

However, it would be appropriate to amplify the sample and follow-up observation to strengthen the statistical power of the results.

## 5. Conclusion

From a motor point of view, the results obtained in Recession and Controlled Myotomy proved to be “functionally” equivalent and in both cases progressive improvements are predictable. In sensory terms, unlike what evidenced for AHP, a total resolution was obtained more frequently in Group  B than in Group  A. Although the Recession technique still remains the most effective and accurate, it is however evident that Controlled Myotomy may prove to be a valid alternate technique both for the fewer intrasurgical risks involved and for the satisfactory results in terms of AHP which may be achieved in time.

## References

[1] Bakunowicz-Łazarczyk A., Urban B., Łazarczyk J. (2003). The surgical results of correcting strabismus with inferior oblique hyperfunction. *Klinika Oczna*.

[2] Arrico L., Migliorini R., Giannotti R., Collini S., Malagola R. (2012). Clinical manifestations due to pharmacological interactions in pediatric ophthalmic surgery: topical drugs and general anaesthesia. *Il Giornale di Chirurgia*.

[3] Malagola R., Arrico L., Migliorini R., D'Ambrosio E. M., Grenga R. (2012). Ocular traumatology in children. A retrospective study. *Il Giornale di Chirurgia*.

[4] Ludwig I. H., Clark R. A., Stager D. R. (2013). New strabismus surgical techniques. *Journal of AAPOS*.

[5] Landolt E. (1885). La tenotomia de loblique inferieure. *Archives of Ophthalmology*.

[6] White M. R. (1943). Recession of the inferior oblique muscle. *Archives of Ophthalmology*.

[7] Pannarale M. R., Leonardi E. (1978). On some techniques of weakening the inferior oblique muscle. *Bollettino di Oculistica*.

[8] Apt L., Call N. B. (1978). Inferior oblique muscle recession. *American Journal of Ophthalmology*.

[9] Yoo J. H., Kim S.-H., Seo J. W., Paik H. J., Cho Y. A. (2013). Self-grading effect of inferior oblique recession. *Journal of Pediatric Ophthalmology and Strabismus*.

[10] Farvardin M., Bagheri M., Pakdel S. (2013). Combined resection and anterior transposition of the inferior oblique muscle for treatment of large primary position hypertropia caused by unilateral superior oblique muscle palsy. *Journal of AAPOS*.

[11] Gregory M. E., Hussin H. M., Dutton G. N. (2011). Inferior oblique recession: an efficient technique. *Strabismus*.

[12] Rajavi Z., Molazadeh A., Ramezani A., Yaseri M. (2011). A randomized clinical trial comparing myectomy and recession in the management of inferior oblique muscle overaction. *Journal of Pediatric Ophthalmology and Strabismus*.

[13] Cruz F. C., Robbins S. L., Kinori M., Acera E. C., Granet D. B. (2015). Z-myotomy of the inferior oblique for small incomitant hypertropias. *Journal of AAPOS*.

[14] Dunlap E. A. (1972). Inferior oblique weakening. Recession, myotomy, myectomy, or disinsertion?. *Annals of Ophthalmology*.

[15] Migliorini R., Collini S., Malagola R., Servidio A., Cannata R., Arrico L. (2015). Anesthesia in the surgery of strabismus: role of anesthetic agents in the ocular deviation and surgical outcome. *Journal of Anesthesia Clinical Research*.

[16] Migliorini R., Comberiati A. M., Galeoto G., Fratipietro M., Arrico L. (2015). Eye motility alterations in retinitis pigmentosa. *Journal of Ophthalmology*.

[17] Niyaz L., Yücel O. E., Gul A. (2016). Infrequent complications of inferior oblique recession surgery. *Seminars in Ophthalmology*.

[18] Parks M. M. (1972). The weakening surgical procedures for eliminating overaction of the inferior oblique muscle. *American Journal of Ophthalmology*.

[19] Parks M. M. (1978). Causes of the adhesive syndrome. Symposium on strabismus. *Transactions of the New Orleans Academy of Ophthalmology*.

[20] Wilson M. E., Parks M. M., Price R. L. (1989). Primary inferior oblique overaction in congenital esotropia, accommodative esotropia, and intermittent exotropia. *Ophthalmology*.

